# Case report – Asterixis Post High Frequency Focused-Ultrasound Thalamotomy

**DOI:** 10.5334/tohm.718

**Published:** 2022-08-30

**Authors:** Ashley Mears, Ali R. Rezai, Richa Tripathi

**Affiliations:** 1 Rockefeller Neuroscience Institute, Morgantown, WV, US; 2 Department of Neurosurgery, West Virginia University, Morgantown, WV, US; 3 Department of Neurology, West Virginia University, Morgantown, WV, US; 4 West Virginia Clinical and Translational Science Institute, West Virginia University, Morgantown, WV, US

**Keywords:** Focused ultrasound thalamotomy, myoclonus, asterixis

## Abstract

**Background:**

High frequency focused ultrasound is used for treatment of essential tremor. Side effects associated with the procedure may resolve over time. We report a case of negative myoclonus, which has not been reported with this procedure.

**Case report:**

A 73-year-old left-handed man underwent focused ultrasound thalamotomy for treatment of essential tremor. Immediately post procedure he was noted to have negative myoclonus in the treated limb. This side effect resolved over the course of 6 months.

**Discussion:**

Although asterixis has been associated with thalamic infarcts in the past, this has not yet been reported in the literature with MRgFUS procedure and is a novel observation. Occupational and physical therapy may be considered to address this side effect. It is important to counsel patients about the rare occurrence of this complication of therapy but also its potential for complete resolution over time.

Essential tremor (ET) is a common neurological disorder characterized by action and postural tremor. This can affect the quality of life of an individual and significantly impair functioning. For medication refractory tremor, several surgical options are available including Deep Brain Stimulation therapy (DBS), gamma knife radiation, as well as Magnetic Resonance Imaging (MRI) guided Focused Ultrasound (MRgFUS) technology.

MRgFUS is an ablative lesioning procedure that was FDA approved for unilateral treatment of ET in 2016 and Parkinson’s Disease tremor in 2018 [1]. Tremor improvements are the result of lesioning within the ventralis intermedius (Vim) nucleus of thalamus. Involvement of surrounding structures may lead to paresthesia in the contralateral fingertips, lips, or tongue, dysarthria or dysphagia, gait imbalance as well as limb ataxia. Most side effects are transient and may improve over time although persistent side effects have been reported in cases even a year after the procedure [1]. Here we report a patient who underwent MRgFUS for ET and developed negative myoclonus as a side effect of the procedure. To our knowledge, this potential side effect has not been reported with this procedure so far.

A 73-year-old left-handed man presented with bilateral progressive hand tremor that started over 15 years ago. He also had head and voice tremor. The left hand tremor interfered with his writing, cutting food, and feeding himself. He was evaluated for MRgFUS surgical intervention for left hand tremor, after failing medical treatment. His left hand tremor was grade 2 on finger to nose testing and grade 4 on archimedes spiral per the essential tremor rating assessment scale (TETRAS) [2]. His skull density ratio was calculated at 0.46 [3]. Prior to the procedure, the remainder of his physical exam showed full strength [5/5 per Medical Research Council (MRC) strength rating], decreased sensation to light touch of the left leg, and 1+ reflexes throughout with absent patellar reflexes bilaterally. Prior to the procedure, he was maintained on primidone 150mg three times a day dose.

During the MRgFUS procedure, seven sonications were performed at 3 targets with an average temperature reaching 59 degrees Celsius and maximum temperature of 66 degrees Celsius. At location X 13.9, Y 6.1, and Z 1.0 (target 1) from the anterior commissure -posterior commissure (AC-PC) line the patient experienced mild ataxia during sonication number 4 with 60–70% improvement in his left-hand tremor. Moving superior by 0.5 mm (target 2), the 5^th^ sonication was performed resulting in nausea and paresthesia in the index, middle and ring fingertips. Tremor improved by 70%. Finally, with a move 0.5 mm anterior (target 3), during the 6^th^ sonication, the patient suffered severe nausea. He noted that the tingling had reduced from the previous sonication. Examination showed 75% improvement in tremor. On the 7^th^ sonication at target 3, the maximum temperature reached was 66 degrees Celsius. Here, the patient noted tingling in the left lip, and a neurologic exam showed 85% tremor improvement from baseline.

Post-procedure, MRI T2 images showed focal targeted lesion in the right thalamus with adjacent edema (See Figure 1). Examination revealed ataxia in left hand and paresthesias of the left hand three digits, and left lower lip. It was noted that the patient now had mild negative myoclonus i.e. asterixis, involving the left shoulder and leg. He was treated with dexamethasone 4 mg every 6 hours during observation for 24 hours. Of note, patient’s medication list included atorvastatin, lisinopril, and primidone which were unlikely to cause asterixis. Serum metabolic panel, ammonia levels, liver, and pancreatic function tests were obtained and were within normal limits.

**Figure 1:**
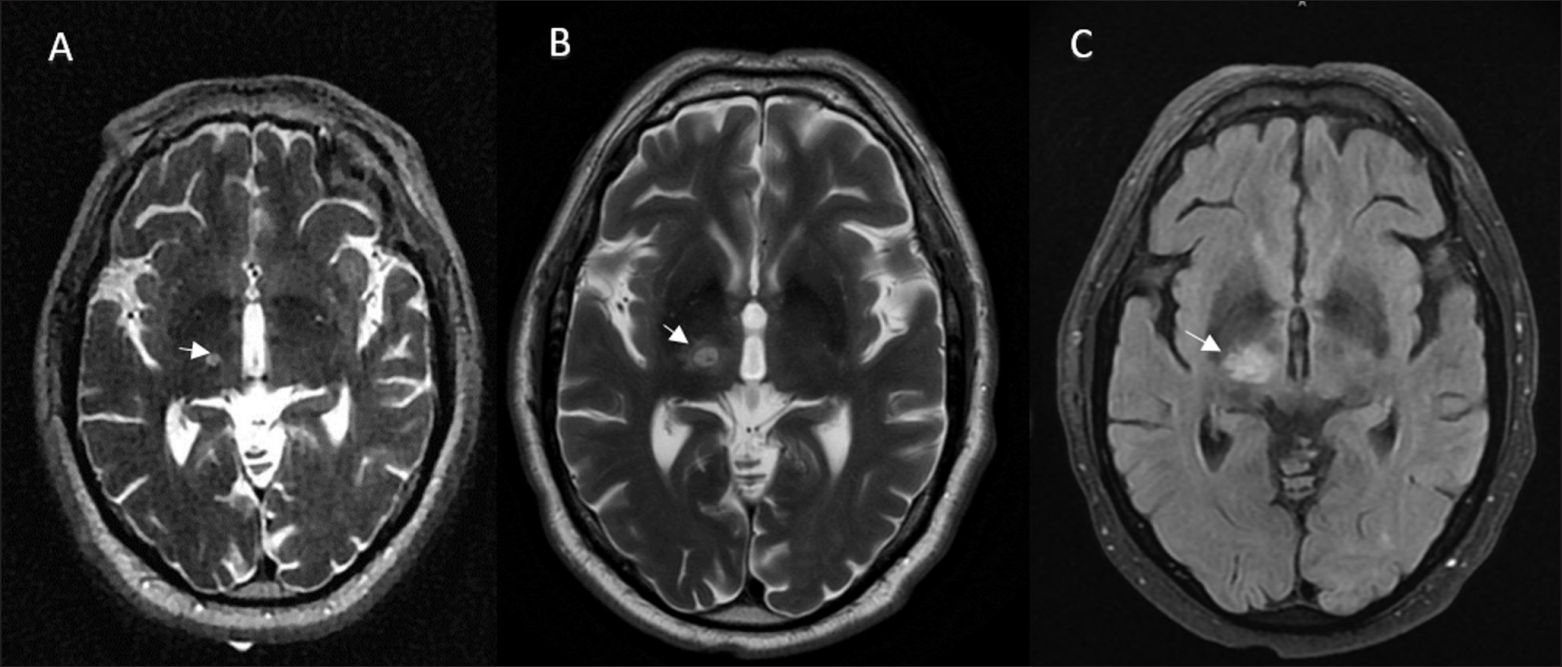
Left to right A. Postoperative day-0 (POD -0) – transverse T2 image with lesion in the right thalamus. B. POD-1 expansion of lesion seen on T2 sequence. C. POD-1, Fluid-attenuated inversion recovery (FLAIR) sequence demonstrating edema encroaching posterior limb of internal capsule.

24 hours post-procedure, patient exhibited significant asterixis in the left upper and lower extremities with ataxia in the left hand. Strength in left shoulder adduction was 4/5, shoulder abduction 4/5, external rotation 3/5, elbow flexion 4/5, elbow extension 4/5, left hip flexion 4/5, knee extension 4/5, knee flexion 4/5 and dorsiflexion 4/5 per MRC grading. Patient experienced post-procedural imbalance related to the MRgFUS procedure, which was corrected using a walker. A repeat MRI demonstrated an interval increase in the surrounding edema but also noted extension into the internal capsule (Figure 1). The patient was discharged with a 4 mg dexamethasone taper. The patient continued with physical therapy until his next clinic visit.

One week post-procedure, he noted some improvement in imbalance and asterixis. On routine 4-week clinical follow up patient had improved strength in the left upper and lower extremities (4/5 on hip flexion remainder muscle groups were 5/5) and despite having some residual postural instability did not use ambulatory devices. The patient reported subjective improvement of his left-hand symptoms by at least 50%, which was confounded by the presence of asterixis (See video). Paresthesias on the left side of his lips and the left side of the tongue persisted. He also reported additional taste disturbances that started a few days after the procedure. He restarted topiramate 50 mg twice a day dose for management of tremor of his contralateral limb. He continued to participate in physical therapy.

**Video:** **Neurological exam pre and post procedure.** Pre-operative exam displaying postural (wing beating posture) tremor in bilateral hands and tremor on drawing spirals. No asterixis seen during this exam. 1- month follow-up shows trace asterixis on dorsiflexion of the left hand along with dysmetria on finger to nose exam. By 6 month follow-up no asterixis was seen on exam and improvement in dysmetria which was previously noted.

At 6 months follow up he reported that the paresthesias in his left lip and tongue had improved by 60% from the onset and that asterixis had resolved completely. Overall, at 6 months he was happy with improvements in his left-hand tremor, which was now 90% subjectively improved per patient and graded 0–1 on individual scoring of action tremor, kinetic tremor and drawing spirals per TETRAS score. He was weaned off primidone and topiramate by then and was not on any tremor medication as this was not bothersome.

About 1-year post-procedure, patient’s balance and strength was back to pre-procedure baselines. He described 80% subjective improvement in the lip paresthesias and stated it was no longer bothersome. His left hand postural and action tremor was 0 but spiral was graded as 3 per TETRAS.

Although asterixis has been associated with thalamic infarcts in the past this has not been reported in literature with MRgFUS procedure yet [4]. Asterixis is a manifestation of intermittent failure in maintaining sustained muscle contraction and tonic posture which is mediated through rubrospinal, reticulospinal and vestibulospinal pathways. Ventrolateral (VL) nuclei of thalamus controls these fibers with further connections to cerebellorubral tracts. Strokes along these pathways including the VL nuclei have been associated with contralateral asterixis as seen in our case [4,5].

Our patient’s asterixis caused him functional disability early on but resolved completely by 6 months post-therapy. Depending on the severity and extent of limb or body part involvement, patients may require additional assistance with the use of a walker and ongoing physical as well as occupational therapy treatments, as demonstrated in our patient. Evaluation by therapy is of great utility in such instances as this patient responded very well to regular treatments. Asterixis and other rare but possible side effects may require continued monitoring to ensure the safety of the individual.
